# Longitudinal Melanonychia as a Presenting Sign of Onychopapilloma: A Case Report and Review of the Literature

**DOI:** 10.1155/crdm/3185082

**Published:** 2026-04-29

**Authors:** Erandy Alicia Salcedo Elguea, Lizet Valles Montaño, Anna Karina Soto Posada, Jorge Alberto Guerra Villalobos

**Affiliations:** ^1^ Internal Medicine Resident, ISSSTE General Hospital, Juarez City, Chihuahua, Mexico; ^2^ Dermatology Department, IMSS General Zone Hospital Number 6, Juarez City, Chihuahua, Mexico

**Keywords:** dermoscopy, histological techniques, nail diseases, subungual neoplasms

## Abstract

Onychopapilloma is a benign tumor of the nail unit characterized by broad clinical variability, which frequently delays diagnosis. It typically presents as longitudinal erythronychia, melanonychia, subungual hyperkeratosis, or a distal nail mass. We report a 65‐year‐old woman with a chronic single‐digit nail lesion initially misdiagnosed as onychomycosis. Dermoscopy revealed distal hyperkeratosis and a subungual mass. Histopathological examination confirmed onychopapilloma, and complete surgical excision resulted in symptom resolution. This report underscores the importance of distinguishing this entity from malignant nail tumors and highlights the diagnostic value of dermoscopy and histopathology. Early recognition may prevent diagnostic delay and unnecessary treatments.

## 1. Introduction

Onychopapilloma is a benign tumor of the nail unit originating from the distal matrix or nail bed. It may present in various forms, including longitudinal melanonychia, erythronychia, leukonychia, or subungual hyperkeratosis [1–5]. This diversity of manifestations, together with limited clinical familiarity, contributes to frequent underdiagnosis, as the condition is often mistaken for onychomycosis or chronic traumatic nail changes [2, 3, 6].

Dermoscopy and high‐frequency ultrasound allow more precise lesion characterization; however, definitive diagnosis relies on histopathological examination [1]. Despite its benign nature, onychopapilloma is clinically relevant because it may mimic malignant tumors such as melanoma or nail‐unit squamous cell carcinoma, particularly when pigmentation or progressive growth is present [5, 6].

We describe a representative case of onychopapilloma presenting as longitudinal melanonychia and provide clinical, dermoscopic, and histopathological correlation supported by the recent literature.

## 2. Case Presentation

A 65‐year‐old woman with a history of systemic hypertension and breast cancer in remission for 15 years presented with a 2‐year history of a nail lesion involving the left thumb. She had received empiric antifungal treatment on multiple occasions without improvement; no mycologic testing was performed prior to treatment initiation.

**FIGURE 1:**
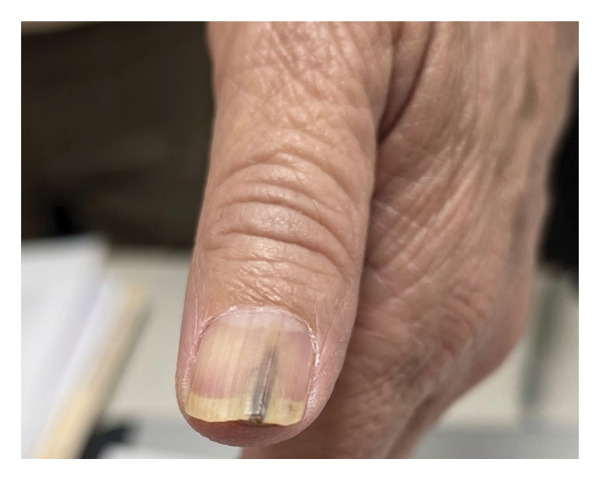
Longitudinal melanonychia band with subungual hyperkeratosis and distal onycholysis of the left thumb.

**FIGURE 2:**
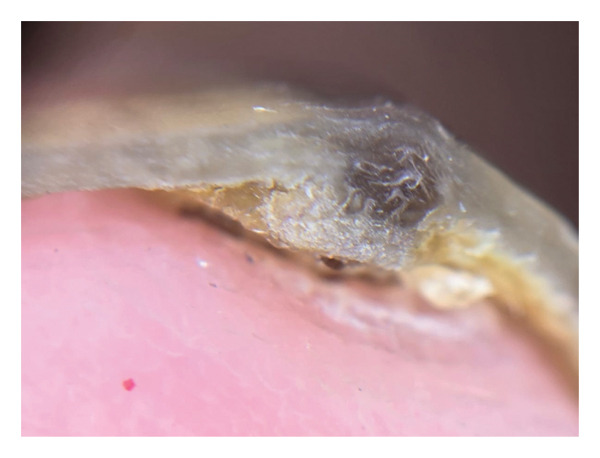
Dermoscopy of the free edge of the nail plate shows a subungual mass and hyperkeratosis.

Upon examination, a monodactyl onychopathy was identified, characterized by subungual hyperkeratosis distributed within a central longitudinal melanonychia band extending from the proximal nail fold to the free edge, with associated yellow discoloration of the lateral nail plate consistent with partial onycholysis, and a distal nail plate growth that was slightly painful on palpation (Figure 1). Dermoscopy of the free edge of the nail plate demonstrated a subungual mass with associated hyperkeratosis (Figure 2). The dermoscopic examination of the nail bed was performed; however, no photographic documentation was obtained. The findings are based on a clinical and free‐edge dermoscopic examination.

**FIGURE 3:**
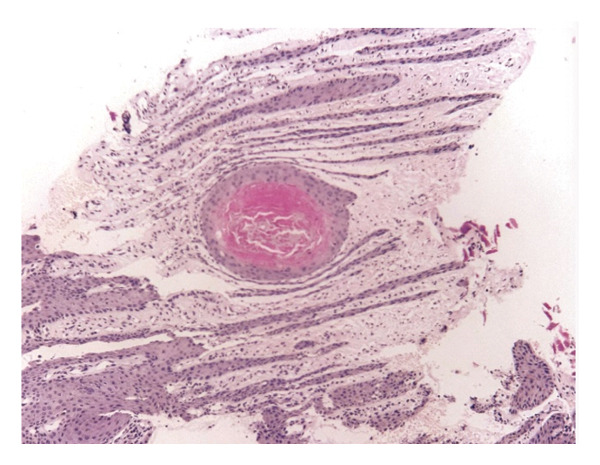
Epithelium with hyperplasia, papillomatosis patterns, and asymmetric elongation of the rete ridges, preserving the integrity of the nail plate, with mitotic figures. Hematoxylin and eosin stain, 4x.

**FIGURE 4:**
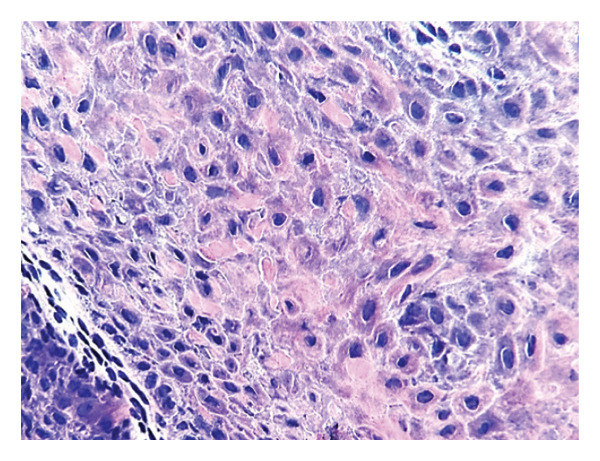
Hypergranulosis, keratinocytes with hyperchromatic nuclei, indented borders, and vacuolar retraction, without findings of malignancy. Hematoxylin and eosin stain, 10x.

Under digital nerve block anesthesia with lidocaine, partial nail plate avulsion was performed followed by shave excision of the nail bed lesion. The histopathological analysis showed hyperplastic epithelium with papillomatosis (Figure 3), hypergranulosis, and keratinocytes with hyperchromatic nuclei without structural atypia (Figure 4). Based on the clinical and histological findings, the diagnosis of onychopapilloma was confirmed. The patient had a favorable outcome, with complete resolution of the pain, adequate healing, and no recurrence during a follow‐up period of 9 months.

## 3. Discussion

Onychopapilloma is a benign tumor that usually affects a single digit, most commonly the thumb or big toe, and occurs in adults without clear sex predilection [1–4]. The etiology remains incompletely understood, although chronic microtrauma and benign epithelial proliferation have been proposed [5].

The classic presentation is longitudinal erythronychia; however, melanonychia, leukonychia, subungual hyperkeratosis, or distal swelling may also occur [1, 4, 5]. Pigmented presentations warrant exclusion of subungual melanoma [7]. Although typically asymptomatic, up to 40% of patients may report localized tenderness [2].

Dermoscopy facilitates recognition of characteristic patterns such as reddish longitudinal bands, splinter hemorrhages, and distal hyperkeratosis [5]. High‐frequency ultrasound has emerged as a complementary tool, demonstrating a focal, well‐defined hyperechoic subungual mass with a minimal Doppler signal [1, 8, 9]. This allows differentiation from other nail neoplasms and may serve as an adjunct for lesion characterization and surgical planning; however, histopathology remains the reference standard when malignancy cannot be excluded.

Histologically, onychopapilloma is characterized by papillomatosis of the nail bed, hyperkeratosis, hypergranulosis, and irregular elongation of the epithelial ridges. Isolated mitotic figures may be observed without evidence of invasion or significant atypia [3]. Immunohistochemical studies further aid differentiation from squamous cell carcinoma or onychomatricoma, particularly through keratin patterns and specific epithelial markers [10].

Differential diagnoses include onychomycosis, chronic trauma, onychomatricoma, nail‐unit squamous cell carcinoma, and melanoma [1]. Nail‐unit squamous cell carcinoma may rarely present as a longitudinal pigmented band and closely mimic benign entities such as onychopapilloma; therefore, biopsy or complete excision should be considered in adult patients with single‐nail lesions showing pain, pigmentation, progressive growth, or other atypical features suggestive of malignancy [5, 6, 11].

An association between multiple onychopapillomas and BAP1 tumor predisposition syndrome (BAP1‐TPDS) has been described; however, genetic screening is not recommended in cases of solitary lesions [12]. The treatment of choice is complete surgical excision, which serves both diagnostic and therapeutic purposes [2, 6]. Recurrence rates after surgical excision have been reported at approximately 20%, with higher rates observed following tangential compared with longitudinal excision [1, 2]. Alternative therapies, such as pulsed dye laser, have been described in small series, although current evidence remains limited and does not replace excision in most cases [13].

The relationship between onychopapilloma and human papillomavirus (HPV) infection remains uncertain. Unlike nail‐unit squamous cell carcinoma, in which HPV has been detected in up to 70% of cases, a consistent association between HPV and benign onychopapilloma has not been established, and current evidence does not support a causal role; therefore, routine HPV testing is not recommended in the workup of onychopapilloma [4, 11]. HPV evaluation was not performed in the present case.

In our patient, the chronic course and lack of response to empirical treatment prompted re‐evaluation, a diagnostic delay consistent with recent series in which onychopapilloma is frequently misinterpreted as onychomycosis or traumatic nail disease [2, 3]. The coexistence of distal hyperkeratosis, subungual swelling, and longitudinal melanonychia corresponded to clinical patterns previously described in the literature [4]. Because longitudinal melanonychia in older adults warrants careful evaluation to exclude melanoma and nail‐unit squamous cell carcinoma, a diagnostic excision was appropriate and aligned with the current recommendations.

Histopathological examination demonstrated classic features of onychopapilloma. Although mitotic figures were identified, the absence of invasion or significant architectural atypia supported benign epithelial hyperplasia. Complete symptom resolution following the excision confirms the diagnostic and therapeutic value of the procedure.

This case highlights the importance of considering onychopapilloma in persistent single‐digit nail lesions of uncertain etiology and underscores the need to integrate clinical, dermoscopic, and histopathological findings to avoid misdiagnosis and exclude malignant neoplasms. The key characteristics of the published cases and series discussed in this review are summarized in Table 1.

**TABLE 1:** Summary of published cases and series of onychopapilloma included in the literature review.

Reference	Study type	*N*	Age/sex	Digit	Phenotype	Dermoscopy	Ultrasound	Histologic hallmarks	Management	Follow‐up/recurrence
Yun et al., [2]	Case series	50	Adults, F > M	Fingers > toes	Erythronychia, subungual hyperkeratosis, distal mass	Reddish band, splinter hemorrhages, distal hyperkeratosis	NR	Papillomatosis, hypergranulosis, hyperkeratosis	Excision	Low recurrence rate
Mansouri et al., [3]	Case report	1	35 y, M	Right thumb	Longitudinal erythronychia with distal splinter hemorrhages	Pink band 1.7 mm interrupting lunula, filiform hemorrhages; free edge: subungual keratotic mass	NR	Papillomatous epithelial lining, epidermal metaplasia, multinucleated keratinocytes without cytonuclear atypia	Partial avulsion + total excision	NR
Kim et al., [4]	Case series	NR	Adults	Fingers	Erythronychia, leukonychia, melanonychia	Longitudinal band, hyperkeratosis	NR	Papillomatosis, hypergranulosis	Excision	NR
Park [5]	Case report	1	Adult, F	Finger	Longitudinal erythronychia	Reddish band, distal hyperkeratosis	NR	Papillomatosis, hyperkeratosis	Excision	No recurrence
Arias‐Rodríguez et al., [6]	Case report	1	Adult	Finger	Subungual hyperkeratosis, distal mass	NR	NR	Papillomatosis	Excision	NR
Mattioli et al., [1]	Retrospective observational	6	Mean 55.5 y (23–75); 4F, 2M	Fingers/toes	Erythronychia [3], melanonychia [2], splinter hemorrhages [1]	Erythronychia, melanonychia, splinter hemorrhages	Hyperechoic distal mass (5/6); nail bed dishomogeneity (3/6); no Doppler (6/6)	Papillomatosis, acanthosis, matrix metaplasia; large eosinophilic keratinocytes	Diagnostic context (US study)	NR
Canella et al., [8]	Case series	43	Adults	Fingers/toes	Variable	NR	Well‐defined hyperechoic subungual mass	NR	NR	NR
Sechi et al., [9]	Case series	NR	Adults	Fingers/toes	Variable	NR	Focal subungual mass (high/ultra‐high frequency)	NR	NR	NR
Liu et al., [10]	Retrospective single‐center	11	Mean 30.7 y (13–54); 6F, 5M	Thumb (6/11)	Erythronychia [6], melanonychia [3], leukonychia [2]; all with subungual hyperkeratosis	Asymmetric keratotic mass, lamellar keratinization; splinter hemorrhages in some cases	NR	Papillomatosis ± acanthosis, matrix metaplasia; IHC: HK31+, HK75+, K6/K16+; HK34/85/86‐	Excision	NR
Lebensohn et al., [11]	Case series	Multiple	Adults	Multiple digits	Multiple onychopapillomas	NR	NR	Papillomatosis	Excision + genetic screening	NR
Fan et al., [12]	Case series	13	Adults	Fingers	Erythronychia, hyperkeratosis	NR	NR	Papillomatosis	Pulsed dye laser	Improvement, limited follow‐up

*Note:* F: female; M: male; IHC: immunohistochemistry; US: ultrasound.

Abbreviations: NR, not reported; SCC, squamous cell carcinoma.

## Funding

The authors thank the General Hospital IMSS for supporting the preparation of this report and declare that this work was carried out using their own resources. This study did not receive any financial support or funding.

## Consent

No written consent has been obtained from the patients as there are no patient identifiable data included in this case report.

## Conflicts of Interest

The authors declare no conflicts of interest.
